# Crystal structure and Hirshfeld surface analysis of di­iodido­{*N*′-[(*E*)-(phen­yl)(pyridin-2-yl-κ*N*)methylidene]pyridine-2-carbohydrazide-κ^2^ *N*′,*O*}cadmium(II)

**DOI:** 10.1107/S2056989019008831

**Published:** 2019-06-25

**Authors:** Zeliha Atioğlu, Farhad Akbari Afkhami, Mehmet Akkurt, Ali Akbar Khandar, Duane Choquesillo-Lazarte

**Affiliations:** a İlke Education and Health Foundation, Cappadocia University, Cappadocia Vocational College, The Medical Imaging Techniques Program, 50420 Mustafapaşa,Ürgüp, Nevşehir, Turkey; b Department of Inorganic Chemistry, Faculty of Chemistry, University of Tabriz, Tabriz, Iran; c Department of Physics, Faculty of Sciences, Erciyes University, 38039 Kayseri, Turkey; d Laboratorio de Estudios Cristalograficos, IACT, CSIC-Universidad de Granada, Av. De las Palmeras 4, E-18100 Armilla, Granada, Spain

**Keywords:** crystal structure, *N*′-[(*E*)-(pyridin-2-yl)methyl­idene]pyridine-2-carbohydrazide, cadmium, iodide, hydrogen bonding, Hirshfeld surface analysis

## Abstract

In each of the two independent mol­ecules in the asymmetric unit of the title compound, [CdI_2_(C_18_H_14_N_4_O)], the *N*,*O*,*N*′-tridentate *N*′-[(*E*)-(phen­yl)(pyridin-2-yl-κ*N*)methyl­idene]pyridine-2-carbohydrazide ligand and two iodide anions form an I_2_N_2_O penta­coordination sphere, with a distorted square-pyramidal geometry, with an I atom in the apical position. Both mol­ecules feature an intra­molecular N—H⋯N hydrogen bond. In the crystal, weak aromatic π–π stacking inter­actions [centroid–centroid separation = 3.830 (2) Å] link the mol­ecules into dimers.

## Abstract 2

The title compound contains two mol­ecules in the asymmetric unit: both feature a distorted square-pyramidal CdN_2_OI_2_ coordination polyhedron and an intra­molecular N—H⋯N hydrogen bond.

## Chemical context   

Hydrazone ligands show high efficiency in chelating transition-metal ions (Afkhami *et al.*, 2017*a* ▸); such ligands obtained from pyridine carb­oxy­lic acids can act as ditopic ligands because of their two different donor sites, including an N-donor pyridine group and a tridentate coordination pocket, and have the potential to form mono-, di- and multinuclear structures (Afkhami *et al.*, 2017*b* ▸). In this work, we report the synthesis, crystal structure and Hirshfeld surface analysis of the title Cd^II^ complex, (I), containing the tridentate hydrazone ligand *N*′-[(*E*)-(pyridin-2-yl)methyl­idene]pyridine-2-carbohydrazide.
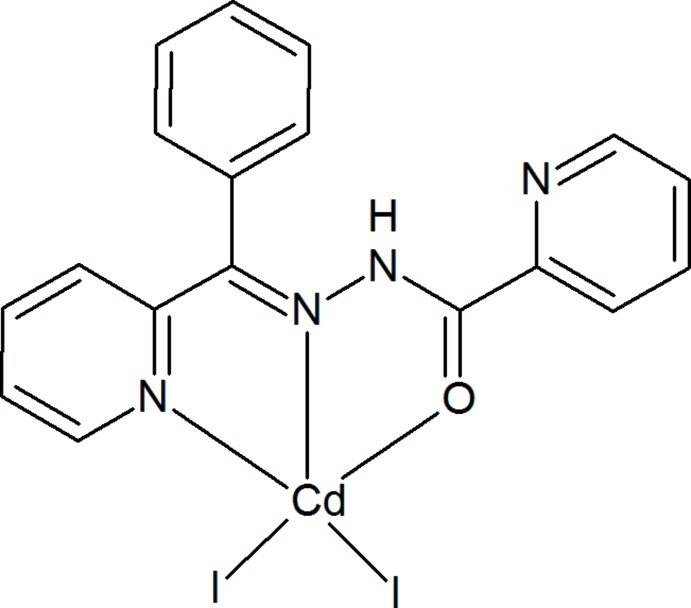


## Structural commentary   

**Figure 1:**
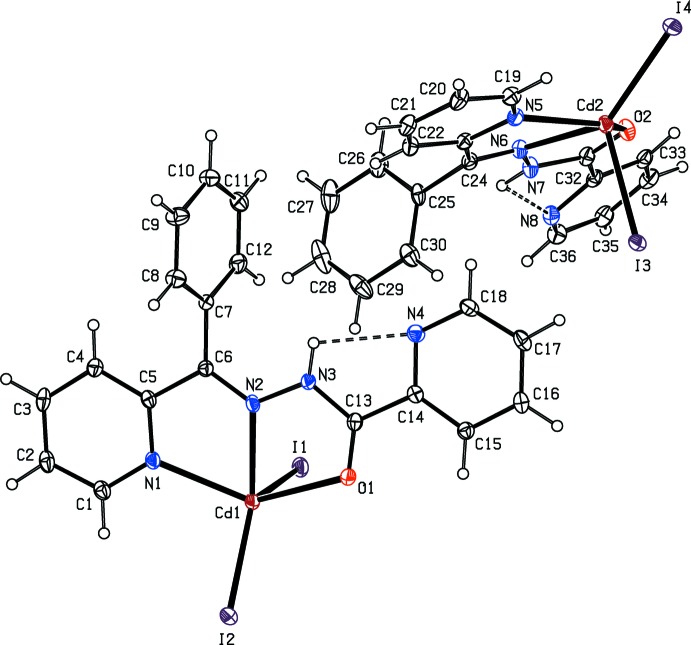
The mol­ecular structure of (I) with displacement ellipsoids drawn at the 30% probability level.

**Figure 2:**
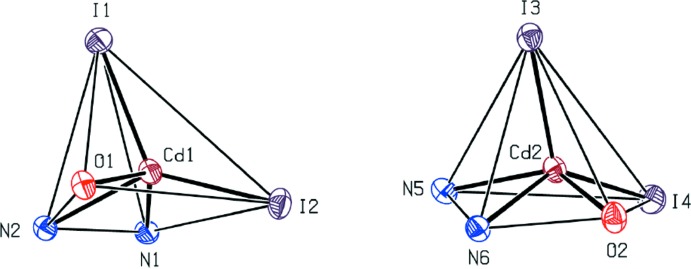
View of the coordination polyhedra about the Cd atoms in (I), showing their distorted square-based pyramidal geometries.

The mol­ecular structure of (I), which contains two [CdI_2_(C_18_H_14_N_4_O)] mol­ecules, *A* (with Cd1) and *B* (with Cd2), in the asymmetric unit is shown in Fig. 1 ▸. In both mol­ecules, the Cd atom is penta­coordinated to two N, one O and two I atoms (Table 1 ▸). The Addison τ parameter (Addison *et al.*, 1984 ▸) qu­anti­fies the distinction between trigonal–bipyramidal (ideally τ = 1) and square-pyramidal (ideally τ = 0) geometries. For the title complex, τ = 0.11 for *A* and 0.09 for *B*, indicating a distorted square-pyramidal geometry (Fig. 2 ▸).

The dihedral angles between the planes of the pyridine rings are 4.44 (17)° for *A* and and 15.63 (17)° for *B*. For *A*, the Cd1—I1 and Cd1—I2 bond lengths are 2.7509 (4) and 2.6917 (4) Å, respectively, and the Cd1—N2 bond length is 2.342 (3) Å. For *B*, the Cd2—I3 and Cd2—I4 bond lengths are 2.7530 (4) and 2.6732 (4) Å, respectively, and the Cd2—N6 bond length is 2.344 (3) Å. Both mol­ecules feature an intra­molecular N—H⋯N hydrogen bond with the pyridine-ring N atom as the acceptor (Table 2 ▸).

## Supra­molecular features and Hirshfeld surface analysis   

**Figure 3:**
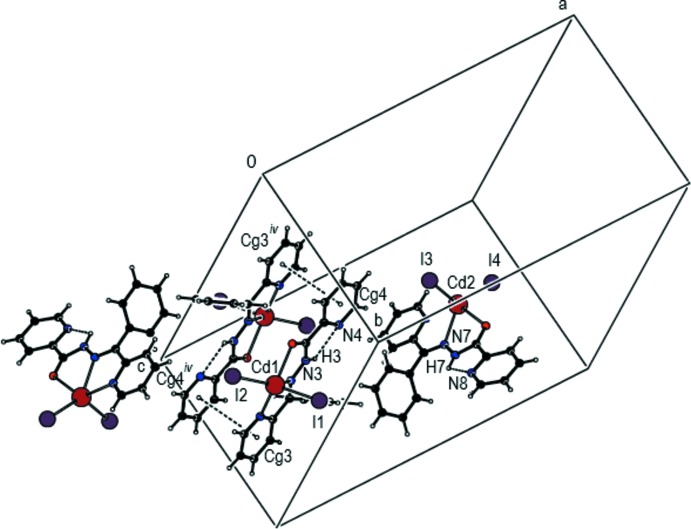
Partial packing diagram of (I) showing the *A* and *B* mol­ecules linked by a pair of π–π stacking inter­actions. Symmetry operation: (iv) −*x*, 1 − *y*, 1 − *z*.

In the crystal of (I), mol­ecules are linked into dimeric *A* + *B* associations by aromatic π–π stacking inter­actions [*Cg*3⋯*Cg*4(−*x*, 1 − *y*, 1 − *z*) = 3.830 (2) Å, where *Cg*3 and *Cg*4 are the centroids of the pyridine rings (N1/C1–C5) and (N4/C14–C18), respectively] (Fig. 3 ▸).

**Figure 4:**
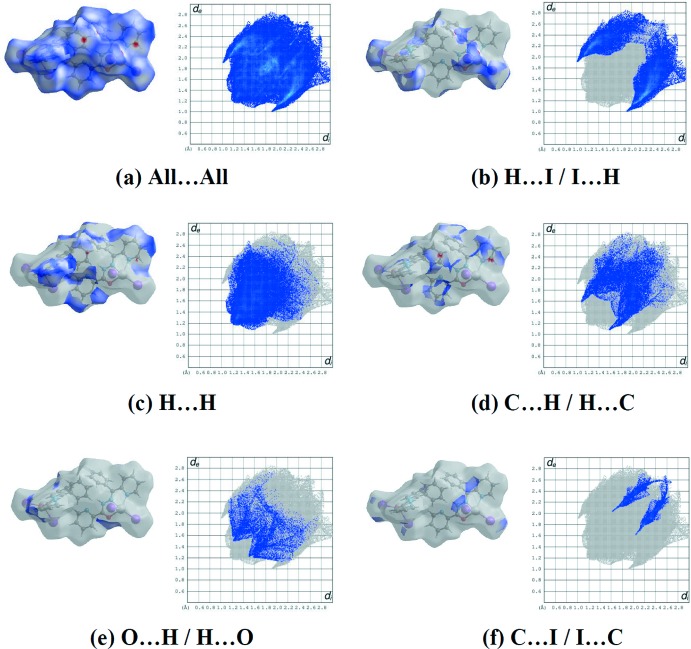
Hirshfeld surface representations and the two-dimensional fingerprint plots for (I), showing (*a*) all inter­actions, and delineated into (*b*) H⋯I/I⋯H, (*c*) H⋯H, (*d*) C⋯H/H⋯C, (*e*) H⋯O/O⋯H and (*f*) C⋯I/I⋯C inter­actions [*d*_e_ and *d*_i_ represent the distances from a point on the Hirshfeld surface to the nearest atoms outside (external) and inside (inter­nal) the surface, respectively].

The Hirshfeld surface analysis (Spackman & Jayatilaka, 2009 ▸) of (I) was performed using *CrystalExplorer3.1* (Wolff *et al.*, 2012 ▸) to generate *d*_norm_ surface plots and two -dimensional fingerprint plots (Spackman & McKinnon, 2002 ▸). Fig. 4 ▸ *a* shows the overall two-dimensional fingerprint plot for the contacts contributing to the Hirshfeld surface. The percentage contributions and Hirshfeld surfaces for H⋯I /I⋯H contacts (30.5%), H⋯H (29.5%), C⋯H / H⋯C (13.3%), H⋯O / O⋯H (5.6%) and C⋯I / I⋯C (4.9%) are shown in Figs. 4 ▸ *b*–*f*, respectively. The full list of percentage surface contributions in given in Table 3 ▸.

## Database survey   

All bond lengths and angles in (I) fall within their expected ranges and are comparable with those reported for related structures, such as bis­{*N*′-[(*E*)-4-hy­droxy­benzyl­idene]-pyridine-4-carbohydrazide-κ*N* ^1^}di­iodido­cadmium methanol disolvate (CCDC refcode: DADHIC; Afkhami *et al.*, 2017*c* ▸), di­bromido­{*N*′-[1-(pyridin-2-yl)ethyl­idene]picolinohydrazide-κ^2^ *N*′,*O*}cadmium (ACUDOT;Akkurt *et al.*, 2012 ▸), di-μ-chlorido-bis­(chlorido­{*N*′-[phen­yl(pyridin-2-yl-κ*N*)methyl­idene]pyridine-2-carbohydrazide-κ^2^ *N*′,*O*}cadmium) (JOBTEB; Akkurt *et al.*, 2014 ▸), bis­{2-[(2,4-di­methyl­phen­yl)imino­meth­yl]pyridine-κ^2^ *N*,*N*′}bis­(thio­cyanato-κ*N*)cadmium (GARTAW; Malekshahian *et al.*, 2012 ▸) and *cis*-di­aqua­bis-[(*E*)-4-(2-hy­droxy­benzyl­idene­amino)­benzoato-κ^2^ *O*,*O*′]cadmium (WEH­SOS; Yao *et al.*, 2006 ▸) in which layers are built by strong O—H⋯O hydrogen bonds. In the crystal of di­iodido-{*N*-[(pyrid­in-2-yl-κ*N*)methyl­idene]picolinohydrazide-κ^2^ *N*′,*O*}cadmium (W­ASCEB; Khandar *et al.*, 2017 ▸), the mol­ecules are linked by N—H⋯I hydrogen bonds, forming chains propagating along [010].

## Synthesis and crystallization   

The *N*′-[(*E*)-(pyridin-2-yl)methyl­idene]pyridine-2-carbohydrazide ligand was synthesized according to the literature method (Abedi *et al.*, 2016 ▸). To prepare single crystals of (I), an equimolar mixture (1.0 mmol) of the hydrazone ligand and metal salt [CdI_2_] were placed in the main arm of a branched tube, and methanol was carefully added to fill the arms (Khandar *et al.*, 2015 ▸). The tube was sealed and the mixture-containing arm was immersed in an oil bath at 333 K while the branched arm was kept at room temperature. After a couple of days, yellow prisms of (I) had been deposited in the cooler arm and these were isolated, filtered off, washed with diethyl ether and dried over P_4_O_10_ *in vacuo*.

## Refinement   

Crystal data, data collection and structure refinement details are summarized in Table 4 ▸. H atoms were placed in calculated positions (C—H = 0.95 Å, N—H= 0.88 Å) and included in the refinement in the riding-model approximation, with *U*_iso_(H) = 1.2*U*_eq_(N,C).

## Supplementary Material

Crystal structure: contains datablock(s) I, global. DOI: 10.1107/S2056989019008831/hb7823sup1.cif

Structure factors: contains datablock(s) I. DOI: 10.1107/S2056989019008831/hb7823Isup2.hkl

CCDC reference: 1935658

Additional supporting information:  crystallographic information; 3D view; checkCIF report

## Figures and Tables

**Table 1:** Selected bond lengths (Å)

Cd1—N2	2.342 (3)	Cd2—N6	2.344 (3)
Cd1—N1	2.380 (3)	Cd2—N5	2.369 (3)
Cd1—O1	2.450 (2)	Cd2—O2	2.481 (3)
Cd1—I2	2.6917 (4)	Cd2—I4	2.6732 (4)
Cd1—I1	2.7509 (4)	Cd2—I3	2.7530 (4)

**Table 2:** Hydrogen-bond geometry (Å, °)

*D*—H⋯*A*	*D*—H	H⋯*A*	*D*⋯*A*	*D*—H⋯*A*
N3—H3⋯N4	0.88	2.27	2.629 (4)	104
N7—H7⋯N8	0.88	2.28	2.640 (4)	104

**Table 3:** Percentage contributions of inter­atomic contacts to the Hirshfeld surface for (I)

Contact	Percentage contribution
H⋯I/I⋯H	30.5
H⋯H	29.5
C⋯H/H⋯C	13.3
H⋯O/O⋯H	5.6
C⋯I/I⋯C	4.9
C⋯C	3.3
N⋯H/H⋯N	2.9
C⋯N/N⋯C	2.8
H⋯Cd/Cd⋯H	2.1
N⋯I/I⋯N	1.7
N⋯O/O⋯N	1.5
I⋯I	0.6
N⋯N	0.4
C⋯O/O⋯C	0.4
C⋯Cd/Cd⋯C	0.3
N⋯Cd/Cd⋯N	0.2

**Table 4:** Experimental details

Crystal data	
Chemical formula	[CdI_2_(C_18_H_14_N_4_O)]
*M*_r_	668.53
Crystal system, space group	Monoclinic, *P*2_1_/*c*
Temperature (K)	100
*a*, *b*, *c* (Å)	19.9158 (15), 11.7252 (9), 17.8349 (14)
β (°)	104.207 (1)
*V* (Å^3^)	4037.4 (5)
*Z*	8
Radiation type	Mo *K*α
μ (mm^−1^)	4.16
Crystal size (mm)	0.34 × 0.33 × 0.17

Data collection	
Diffractometer	Bruker SMART APEX
Absorption correction	Multi-scan (*SADABS*; Bruker, 2009 ▸)
*T*_min_, *T*_max_	0.600, 0.746
No. of measured, independent and observed [*I* > 2σ(*I*)] reflections	24216, 9018, 7948
*R*_int_	0.020
(sin θ/λ)_max_ (Å^−1^)	0.664

Refinement	
*R*[*F* ^2^ > 2σ(*F* ^2^)], *wR*(*F* ^2^), *S*	0.027, 0.064, 1.03
No. of reflections	9018
No. of parameters	469
H-atom treatment	H-atom parameters constrained
Δρ_max_, Δρ_min_ (e Å^−3^)	1.26, −0.56

Computer programs: *APEX2* and *SAINT* (Bruker, 2009 ▸), *SHELXS97* (Sheldrick, 2008 ▸), *SHELXL2014* (Sheldrick, 2015 ▸), *ORTEP-3 for Windows* (Farrugia, 2012 ▸) and *PLATON* (Spek, 2015 ▸).

## supplementary crystallographic information

### Crystal data

**Table:**

[CdI_2_(C_18_H_14_N_4_O)]	*F*(000) = 2496
*M**_r_* = 668.53	*D*_x_ = 2.200 Mg m^−^^3^
Monoclinic, *P*2_1_/*c*	Mo *K*α radiation, λ = 0.71073 Å
*a* = 19.9158 (15) Å	Cell parameters from 9930 reflections
*b* = 11.7252 (9) Å	θ = 2.2–27.8°
*c* = 17.8349 (14) Å	µ = 4.16 mm^−^^1^
β = 104.207 (1)°	*T* = 100 K
*V* = 4037.4 (5) Å^3^	Hexagonal prism, yellow
*Z* = 8	0.34 × 0.33 × 0.17 mm

### Data collection

**Table:**

Bruker SMART APEX diffractometer	7948 reflections with *I* > 2σ(*I*)
Graphite monochromator	*R*_int_ = 0.020
φ and ω scans	θ_max_ = 28.2°, θ_min_ = 2.0°
Absorption correction: multi-scan (SADABS; Bruker, 2009)	*h* = −26→25
*T*_min_ = 0.600, *T*_max_ = 0.746	*k* = −15→12
24216 measured reflections	*l* = −20→22
9018 independent reflections	

### Refinement

**Table:**

Refinement on *F*^2^	Primary atom site location: structure-invariant direct methods
Least-squares matrix: full	Hydrogen site location: inferred from neighbouring sites
*R*[*F*^2^ > 2σ(*F*^2^)] = 0.027	H-atom parameters constrained
*wR*(*F*^2^) = 0.064	*w* = 1/[σ^2^(*F*_o_^2^) + (0.0306*P*)^2^ + 5.7109*P*] where *P* = (*F*_o_^2^ + 2*F*_c_^2^)/3
*S* = 1.03	(Δ/σ)_max_ = 0.001
9018 reflections	Δρ_max_ = 1.26 e Å^−^^3^
469 parameters	Δρ_min_ = −0.56 e Å^−^^3^
0 restraints	

### Special details

**Table:**

Geometry. All esds (except the esd in the dihedral angle between two l.s. planes) are estimated using the full covariance matrix. The cell esds are taken into account individually in the estimation of esds in distances, angles and torsion angles; correlations between esds in cell parameters are only used when they are defined by crystal symmetry. An approximate (isotropic) treatment of cell esds is used for estimating esds involving l.s. planes.

### Fractional atomic coordinates and isotropic or equivalent isotropic displacement parameters (Å^2^)

**Table:**

	*x*	*y*	*z*	*U*_iso_*/*U*_eq_	
C1	−0.15988 (18)	0.7689 (3)	0.5679 (2)	0.0265 (8)	
H1	−0.200797	0.761245	0.527529	0.032*	
C2	−0.1651 (2)	0.8147 (3)	0.6379 (2)	0.0296 (8)	
H2	−0.208603	0.839406	0.644720	0.036*	
C3	−0.1066 (2)	0.8238 (3)	0.6971 (2)	0.0295 (8)	
H3A	−0.108958	0.855873	0.745277	0.035*	
C4	−0.04377 (18)	0.7855 (3)	0.6858 (2)	0.0245 (7)	
H4	−0.002709	0.789869	0.726250	0.029*	
C5	−0.04221 (17)	0.7409 (3)	0.6145 (2)	0.0218 (7)	
C6	0.02285 (17)	0.6967 (3)	0.59802 (19)	0.0205 (7)	
C7	0.08845 (17)	0.6963 (3)	0.65920 (19)	0.0210 (7)	
C8	0.09709 (19)	0.6246 (3)	0.7225 (2)	0.0292 (8)	
H8	0.060605	0.575262	0.727562	0.035*	
C9	0.1594 (2)	0.6251 (4)	0.7788 (2)	0.0352 (9)	
H9	0.165419	0.575842	0.822230	0.042*	
C10	0.2129 (2)	0.6974 (4)	0.7716 (2)	0.0342 (9)	
H10	0.255585	0.697081	0.809828	0.041*	
C11	0.2035 (2)	0.7697 (4)	0.7086 (2)	0.0339 (9)	
H11	0.239690	0.820040	0.703963	0.041*	
C12	0.1420 (2)	0.7691 (3)	0.6523 (2)	0.0306 (8)	
H12	0.136162	0.818266	0.608864	0.037*	
C13	0.06053 (17)	0.5883 (3)	0.4289 (2)	0.0214 (7)	
C14	0.12412 (17)	0.5528 (3)	0.4049 (2)	0.0216 (7)	
C15	0.11899 (18)	0.5094 (3)	0.3316 (2)	0.0242 (7)	
H15	0.075311	0.501950	0.295562	0.029*	
C16	0.17994 (19)	0.4772 (3)	0.3122 (2)	0.0283 (8)	
H16	0.178904	0.447717	0.262261	0.034*	
C17	0.24142 (18)	0.4888 (3)	0.3667 (2)	0.0304 (8)	
H17	0.283676	0.466404	0.355316	0.036*	
C18	0.24135 (18)	0.5336 (3)	0.4386 (2)	0.0279 (8)	
H18	0.284391	0.541529	0.475719	0.033*	
Cd1	−0.08166 (2)	0.70487 (2)	0.42925 (2)	0.02325 (6)	
I1	−0.02803 (2)	0.89997 (2)	0.38096 (2)	0.02834 (6)	
I2	−0.20450 (2)	0.60932 (2)	0.36168 (2)	0.03106 (7)	
N1	−0.09966 (14)	0.7350 (3)	0.55485 (17)	0.0232 (6)	
N2	0.01685 (14)	0.6622 (2)	0.52758 (16)	0.0215 (6)	
N3	0.07222 (14)	0.6208 (3)	0.50403 (16)	0.0226 (6)	
H3	0.113503	0.615314	0.535951	0.027*	
N4	0.18386 (15)	0.5661 (3)	0.45830 (18)	0.0253 (6)	
O1	0.00248 (12)	0.5878 (2)	0.38410 (14)	0.0256 (5)	
C19	0.47181 (19)	0.5871 (3)	0.5866 (2)	0.0270 (8)	
H19	0.511452	0.549614	0.577806	0.032*	
C20	0.4403 (2)	0.5409 (3)	0.6405 (2)	0.0314 (8)	
H20	0.457550	0.472829	0.667323	0.038*	
C21	0.3830 (2)	0.5955 (3)	0.6547 (2)	0.0303 (8)	
H21	0.359945	0.565392	0.691081	0.036*	
C22	0.36007 (19)	0.6954 (3)	0.6145 (2)	0.0261 (7)	
H22	0.321326	0.735372	0.623582	0.031*	
C23	0.39437 (17)	0.7359 (3)	0.5611 (2)	0.0224 (7)	
C24	0.37348 (17)	0.8435 (3)	0.5184 (2)	0.0225 (7)	
C25	0.30874 (18)	0.9009 (3)	0.5247 (2)	0.0255 (8)	
C26	0.3113 (2)	0.9956 (4)	0.5723 (3)	0.0385 (10)	
H26	0.354632	1.025469	0.599897	0.046*	
C27	0.2505 (3)	1.0457 (4)	0.5790 (3)	0.0543 (13)	
H27	0.251823	1.110396	0.611406	0.065*	
C28	0.1876 (3)	1.0018 (4)	0.5387 (3)	0.0526 (14)	
H28	0.145841	1.036347	0.544022	0.063*	
C29	0.1845 (2)	0.9088 (4)	0.4909 (3)	0.0438 (11)	
H29	0.140978	0.879841	0.462879	0.053*	
C30	0.2457 (2)	0.8577 (4)	0.4840 (3)	0.0350 (9)	
H30	0.244173	0.793135	0.451413	0.042*	
C31	0.43945 (18)	1.0017 (3)	0.3850 (2)	0.0245 (7)	
C32	0.41803 (18)	1.1068 (3)	0.3382 (2)	0.0238 (7)	
C33	0.4640 (2)	1.1575 (3)	0.3007 (2)	0.0269 (8)	
H33	0.508151	1.124917	0.303051	0.032*	
C34	0.4431 (2)	1.2568 (3)	0.2598 (2)	0.0307 (8)	
H34	0.472542	1.294272	0.233131	0.037*	
C35	0.3784 (2)	1.3003 (3)	0.2587 (2)	0.0304 (8)	
H35	0.363169	1.369376	0.232195	0.036*	
C36	0.3361 (2)	1.2419 (3)	0.2967 (2)	0.0294 (8)	
H36	0.291160	1.271413	0.293948	0.035*	
Cd2	0.49190 (2)	0.75304 (2)	0.44240 (2)	0.02348 (6)	
I3	0.39729 (2)	0.65574 (2)	0.32031 (2)	0.02644 (6)	
I4	0.62809 (2)	0.71202 (2)	0.47863 (2)	0.03079 (7)	
N5	0.44979 (15)	0.6813 (3)	0.54638 (17)	0.0241 (6)	
N6	0.41289 (15)	0.8790 (2)	0.47642 (17)	0.0233 (6)	
N7	0.39722 (15)	0.9746 (2)	0.43223 (17)	0.0246 (6)	
H7	0.361299	1.017289	0.434038	0.030*	
N8	0.35512 (15)	1.1468 (3)	0.33692 (18)	0.0266 (6)	
O2	0.49091 (13)	0.9444 (2)	0.38286 (15)	0.0294 (6)	

### Atomic displacement parameters (Å^2^)

**Table:**

	*U*^11^	*U*^22^	*U*^33^	*U*^12^	*U*^13^	*U*^23^
C1	0.0237 (17)	0.030 (2)	0.0272 (19)	0.0041 (15)	0.0095 (15)	0.0072 (15)
C2	0.0316 (19)	0.033 (2)	0.029 (2)	0.0070 (16)	0.0171 (16)	0.0084 (16)
C3	0.041 (2)	0.030 (2)	0.0241 (19)	0.0062 (16)	0.0192 (17)	0.0042 (15)
C4	0.0274 (17)	0.0256 (19)	0.0219 (18)	0.0009 (14)	0.0085 (14)	0.0011 (14)
C5	0.0210 (16)	0.0223 (17)	0.0244 (18)	−0.0003 (13)	0.0101 (14)	0.0030 (14)
C6	0.0209 (16)	0.0217 (17)	0.0199 (17)	−0.0009 (13)	0.0066 (13)	−0.0004 (13)
C7	0.0193 (15)	0.0247 (18)	0.0190 (16)	−0.0002 (13)	0.0049 (13)	−0.0033 (13)
C8	0.0287 (18)	0.029 (2)	0.029 (2)	−0.0017 (15)	0.0043 (15)	0.0055 (15)
C9	0.035 (2)	0.036 (2)	0.030 (2)	−0.0039 (17)	−0.0013 (17)	0.0058 (17)
C10	0.0255 (18)	0.042 (2)	0.032 (2)	−0.0019 (17)	0.0016 (16)	−0.0077 (18)
C11	0.0280 (19)	0.042 (2)	0.035 (2)	−0.0105 (17)	0.0130 (17)	−0.0033 (18)
C12	0.034 (2)	0.036 (2)	0.0242 (19)	−0.0050 (17)	0.0111 (16)	0.0030 (16)
C13	0.0248 (17)	0.0197 (17)	0.0212 (17)	0.0010 (13)	0.0084 (14)	0.0022 (13)
C14	0.0220 (16)	0.0196 (17)	0.0241 (18)	0.0013 (13)	0.0075 (14)	0.0027 (13)
C15	0.0246 (17)	0.0250 (18)	0.0234 (18)	−0.0014 (14)	0.0069 (14)	0.0001 (14)
C16	0.0298 (18)	0.028 (2)	0.031 (2)	0.0020 (15)	0.0164 (16)	0.0002 (16)
C17	0.0232 (17)	0.028 (2)	0.043 (2)	0.0032 (15)	0.0142 (16)	−0.0020 (17)
C18	0.0197 (16)	0.0265 (19)	0.037 (2)	−0.0008 (14)	0.0063 (15)	−0.0052 (16)
Cd1	0.01973 (12)	0.03217 (15)	0.01853 (13)	0.00340 (10)	0.00597 (10)	0.00226 (10)
I1	0.02828 (12)	0.03337 (14)	0.02603 (13)	0.00073 (10)	0.01178 (10)	0.00283 (10)
I2	0.02091 (11)	0.04454 (16)	0.02837 (13)	−0.00209 (10)	0.00728 (9)	−0.00054 (11)
N1	0.0219 (14)	0.0293 (16)	0.0201 (15)	0.0026 (12)	0.0087 (12)	0.0045 (12)
N2	0.0196 (13)	0.0252 (15)	0.0218 (15)	0.0054 (11)	0.0087 (11)	0.0014 (12)
N3	0.0195 (13)	0.0299 (16)	0.0186 (14)	0.0059 (12)	0.0052 (11)	−0.0005 (12)
N4	0.0235 (14)	0.0240 (15)	0.0282 (16)	0.0021 (12)	0.0056 (12)	−0.0024 (12)
O1	0.0227 (12)	0.0337 (14)	0.0206 (12)	0.0040 (10)	0.0057 (10)	−0.0023 (10)
C19	0.0298 (18)	0.0232 (18)	0.0265 (19)	0.0055 (15)	0.0038 (15)	0.0003 (14)
C20	0.044 (2)	0.029 (2)	0.0211 (18)	0.0063 (17)	0.0077 (16)	0.0018 (15)
C21	0.041 (2)	0.030 (2)	0.0222 (19)	0.0011 (16)	0.0106 (16)	0.0025 (15)
C22	0.0300 (18)	0.0263 (19)	0.0239 (18)	0.0039 (15)	0.0100 (15)	0.0004 (15)
C23	0.0252 (17)	0.0218 (17)	0.0198 (17)	0.0017 (14)	0.0046 (14)	−0.0012 (13)
C24	0.0243 (16)	0.0219 (17)	0.0219 (17)	0.0028 (14)	0.0067 (14)	−0.0026 (14)
C25	0.0300 (18)	0.0250 (19)	0.0268 (19)	0.0060 (14)	0.0172 (15)	0.0085 (14)
C26	0.047 (2)	0.031 (2)	0.042 (2)	0.0089 (18)	0.019 (2)	−0.0008 (18)
C27	0.073 (3)	0.042 (3)	0.061 (3)	0.023 (3)	0.042 (3)	0.004 (2)
C28	0.052 (3)	0.046 (3)	0.075 (4)	0.025 (2)	0.045 (3)	0.033 (3)
C29	0.027 (2)	0.048 (3)	0.059 (3)	0.0041 (18)	0.017 (2)	0.027 (2)
C30	0.0312 (19)	0.034 (2)	0.044 (2)	0.0024 (17)	0.0165 (18)	0.0093 (18)
C31	0.0306 (18)	0.0219 (18)	0.0230 (18)	−0.0023 (14)	0.0102 (15)	−0.0020 (14)
C32	0.0310 (18)	0.0215 (18)	0.0196 (17)	−0.0063 (14)	0.0076 (14)	−0.0062 (13)
C33	0.0352 (19)	0.0255 (19)	0.0213 (18)	−0.0065 (15)	0.0098 (15)	−0.0066 (14)
C34	0.043 (2)	0.027 (2)	0.0218 (19)	−0.0086 (17)	0.0082 (16)	−0.0013 (15)
C35	0.042 (2)	0.0246 (19)	0.0251 (19)	0.0006 (16)	0.0099 (17)	0.0026 (15)
C36	0.0334 (19)	0.0263 (19)	0.029 (2)	0.0031 (15)	0.0077 (16)	0.0028 (15)
Cd2	0.02161 (12)	0.02587 (14)	0.02434 (13)	0.00368 (10)	0.00830 (10)	−0.00026 (10)
I3	0.01929 (11)	0.03300 (13)	0.02632 (12)	0.00173 (9)	0.00425 (9)	0.00129 (10)
I4	0.02047 (11)	0.03842 (15)	0.03207 (14)	0.00249 (10)	0.00375 (10)	−0.00371 (10)
N5	0.0261 (15)	0.0262 (16)	0.0203 (15)	0.0034 (12)	0.0061 (12)	0.0000 (12)
N6	0.0266 (15)	0.0211 (15)	0.0233 (15)	0.0016 (12)	0.0086 (12)	0.0016 (12)
N7	0.0315 (15)	0.0195 (15)	0.0266 (16)	0.0056 (12)	0.0143 (13)	0.0052 (12)
N8	0.0280 (15)	0.0262 (16)	0.0277 (16)	−0.0005 (13)	0.0107 (13)	−0.0019 (13)
O2	0.0292 (13)	0.0266 (14)	0.0361 (15)	0.0038 (11)	0.0152 (11)	0.0024 (11)

### Geometric parameters (Å, º)

**Table:**

C1—N1	1.337 (4)	C19—N5	1.331 (5)
C1—C2	1.385 (5)	C19—C20	1.382 (5)
C1—H1	0.9500	C19—H19	0.9500
C2—C3	1.371 (5)	C20—C21	1.384 (5)
C2—H2	0.9500	C20—H20	0.9500
C3—C4	1.389 (5)	C21—C22	1.391 (5)
C3—H3A	0.9500	C21—H21	0.9500
C4—C5	1.381 (5)	C22—C23	1.385 (5)
C4—H4	0.9500	C22—H22	0.9500
C5—N1	1.360 (4)	C23—N5	1.356 (4)
C5—C6	1.491 (5)	C23—C24	1.480 (5)
C6—N2	1.297 (4)	C24—N6	1.280 (5)
C6—C7	1.482 (4)	C24—C25	1.483 (5)
C7—C8	1.385 (5)	C25—C30	1.383 (5)
C7—C12	1.395 (5)	C25—C26	1.391 (6)
C8—C9	1.391 (5)	C26—C27	1.377 (6)
C8—H8	0.9500	C26—H26	0.9500
C9—C10	1.392 (6)	C27—C28	1.382 (8)
C9—H9	0.9500	C27—H27	0.9500
C10—C11	1.385 (6)	C28—C29	1.376 (7)
C10—H10	0.9500	C28—H28	0.9500
C11—C12	1.380 (5)	C29—C30	1.391 (6)
C11—H11	0.9500	C29—H29	0.9500
C12—H12	0.9500	C30—H30	0.9500
C13—O1	1.233 (4)	C31—O2	1.234 (4)
C13—N3	1.357 (4)	C31—N7	1.366 (4)
C13—C14	1.492 (5)	C31—C32	1.491 (5)
C14—N4	1.338 (4)	C32—N8	1.332 (5)
C14—C15	1.384 (5)	C32—C33	1.393 (5)
C15—C16	1.394 (5)	C33—C34	1.382 (5)
C15—H15	0.9500	C33—H33	0.9500
C16—C17	1.371 (5)	C34—C35	1.380 (6)
C16—H16	0.9500	C34—H34	0.9500
C17—C18	1.387 (5)	C35—C36	1.385 (5)
C17—H17	0.9500	C35—H35	0.9500
C18—N4	1.333 (4)	C36—N8	1.330 (5)
C18—H18	0.9500	C36—H36	0.9500
Cd1—N2	2.342 (3)	Cd2—N6	2.344 (3)
Cd1—N1	2.380 (3)	Cd2—N5	2.369 (3)
Cd1—O1	2.450 (2)	Cd2—O2	2.481 (3)
Cd1—I2	2.6917 (4)	Cd2—I4	2.6732 (4)
Cd1—I1	2.7509 (4)	Cd2—I3	2.7530 (4)
N2—N3	1.362 (4)	N6—N7	1.362 (4)
N3—H3	0.8800	N7—H7	0.8800
			
N1—C1—C2	122.7 (3)	N5—C19—C20	123.7 (3)
N1—C1—H1	118.6	N5—C19—H19	118.1
C2—C1—H1	118.6	C20—C19—H19	118.1
C3—C2—C1	119.1 (3)	C19—C20—C21	118.8 (4)
C3—C2—H2	120.4	C19—C20—H20	120.6
C1—C2—H2	120.4	C21—C20—H20	120.6
C2—C3—C4	119.2 (3)	C20—C21—C22	118.5 (4)
C2—C3—H3A	120.4	C20—C21—H21	120.8
C4—C3—H3A	120.4	C22—C21—H21	120.8
C5—C4—C3	118.7 (3)	C23—C22—C21	119.2 (3)
C5—C4—H4	120.7	C23—C22—H22	120.4
C3—C4—H4	120.7	C21—C22—H22	120.4
N1—C5—C4	122.3 (3)	N5—C23—C22	122.3 (3)
N1—C5—C6	115.6 (3)	N5—C23—C24	116.2 (3)
C4—C5—C6	122.0 (3)	C22—C23—C24	121.5 (3)
N2—C6—C7	124.6 (3)	N6—C24—C23	116.3 (3)
N2—C6—C5	115.0 (3)	N6—C24—C25	124.4 (3)
C7—C6—C5	120.5 (3)	C23—C24—C25	119.2 (3)
C8—C7—C12	119.9 (3)	C30—C25—C26	120.3 (4)
C8—C7—C6	120.8 (3)	C30—C25—C24	119.2 (3)
C12—C7—C6	119.3 (3)	C26—C25—C24	120.4 (4)
C7—C8—C9	119.7 (4)	C27—C26—C25	119.5 (4)
C7—C8—H8	120.1	C27—C26—H26	120.2
C9—C8—H8	120.1	C25—C26—H26	120.2
C8—C9—C10	120.3 (4)	C26—C27—C28	120.0 (5)
C8—C9—H9	119.9	C26—C27—H27	120.0
C10—C9—H9	119.9	C28—C27—H27	120.0
C11—C10—C9	119.6 (4)	C29—C28—C27	121.0 (4)
C11—C10—H10	120.2	C29—C28—H28	119.5
C9—C10—H10	120.2	C27—C28—H28	119.5
C12—C11—C10	120.4 (4)	C28—C29—C30	119.3 (4)
C12—C11—H11	119.8	C28—C29—H29	120.4
C10—C11—H11	119.8	C30—C29—H29	120.4
C11—C12—C7	120.1 (4)	C25—C30—C29	119.9 (4)
C11—C12—H12	120.0	C25—C30—H30	120.1
C7—C12—H12	120.0	C29—C30—H30	120.1
O1—C13—N3	123.2 (3)	O2—C31—N7	122.3 (3)
O1—C13—C14	122.7 (3)	O2—C31—C32	124.0 (3)
N3—C13—C14	114.1 (3)	N7—C31—C32	113.7 (3)
N4—C14—C15	124.2 (3)	N8—C32—C33	124.6 (3)
N4—C14—C13	115.7 (3)	N8—C32—C31	116.0 (3)
C15—C14—C13	120.2 (3)	C33—C32—C31	119.3 (3)
C14—C15—C16	117.9 (3)	C34—C33—C32	117.6 (4)
C14—C15—H15	121.1	C34—C33—H33	121.2
C16—C15—H15	121.1	C32—C33—H33	121.2
C17—C16—C15	118.5 (3)	C35—C34—C33	118.6 (4)
C17—C16—H16	120.7	C35—C34—H34	120.7
C15—C16—H16	120.7	C33—C34—H34	120.7
C16—C17—C18	119.4 (3)	C34—C35—C36	119.2 (4)
C16—C17—H17	120.3	C34—C35—H35	120.4
C18—C17—H17	120.3	C36—C35—H35	120.4
N4—C18—C17	123.2 (3)	N8—C36—C35	123.5 (4)
N4—C18—H18	118.4	N8—C36—H36	118.3
C17—C18—H18	118.4	C35—C36—H36	118.3
N2—Cd1—N1	67.60 (9)	N6—Cd2—N5	68.41 (10)
N2—Cd1—O1	67.15 (9)	N6—Cd2—O2	66.77 (9)
N1—Cd1—O1	131.72 (9)	N5—Cd2—O2	133.56 (9)
N2—Cd1—I2	138.47 (7)	N6—Cd2—I4	139.16 (7)
N1—Cd1—I2	98.79 (7)	N5—Cd2—I4	106.26 (7)
O1—Cd1—I2	103.85 (6)	O2—Cd2—I4	99.62 (6)
N2—Cd1—I1	95.38 (7)	N6—Cd2—I3	95.35 (7)
N1—Cd1—I1	109.34 (7)	N5—Cd2—I3	99.61 (7)
O1—Cd1—I1	90.77 (6)	O2—Cd2—I3	95.99 (6)
I2—Cd1—I1	125.873 (12)	I4—Cd2—I3	124.998 (12)
C1—N1—C5	117.8 (3)	C19—N5—C23	117.6 (3)
C1—N1—Cd1	123.8 (2)	C19—N5—Cd2	125.7 (2)
C5—N1—Cd1	116.9 (2)	C23—N5—Cd2	116.4 (2)
C6—N2—N3	121.4 (3)	C24—N6—N7	121.4 (3)
C6—N2—Cd1	121.3 (2)	C24—N6—Cd2	119.5 (2)
N3—N2—Cd1	116.0 (2)	N7—N6—Cd2	116.2 (2)
C13—N3—N2	116.7 (3)	N6—N7—C31	117.4 (3)
C13—N3—H3	121.7	N6—N7—H7	121.3
N2—N3—H3	121.7	C31—N7—H7	121.3
C18—N4—C14	116.8 (3)	C36—N8—C32	116.5 (3)
C13—O1—Cd1	113.1 (2)	C31—O2—Cd2	113.5 (2)
			
N1—C1—C2—C3	−1.4 (6)	N5—C19—C20—C21	−0.9 (6)
C1—C2—C3—C4	−0.9 (6)	C19—C20—C21—C22	−0.6 (6)
C2—C3—C4—C5	1.0 (5)	C20—C21—C22—C23	0.9 (6)
C3—C4—C5—N1	1.1 (5)	C21—C22—C23—N5	0.1 (5)
C3—C4—C5—C6	−179.2 (3)	C21—C22—C23—C24	−178.2 (3)
N1—C5—C6—N2	2.6 (4)	N5—C23—C24—N6	−5.3 (5)
C4—C5—C6—N2	−177.1 (3)	C22—C23—C24—N6	173.1 (3)
N1—C5—C6—C7	−178.6 (3)	N5—C23—C24—C25	173.1 (3)
C4—C5—C6—C7	1.7 (5)	C22—C23—C24—C25	−8.5 (5)
N2—C6—C7—C8	−112.8 (4)	N6—C24—C25—C30	101.6 (4)
C5—C6—C7—C8	68.6 (5)	C23—C24—C25—C30	−76.6 (4)
N2—C6—C7—C12	67.0 (5)	N6—C24—C25—C26	−79.8 (5)
C5—C6—C7—C12	−111.7 (4)	C23—C24—C25—C26	101.9 (4)
C12—C7—C8—C9	−0.4 (6)	C30—C25—C26—C27	0.4 (6)
C6—C7—C8—C9	179.4 (4)	C24—C25—C26—C27	−178.1 (4)
C7—C8—C9—C10	0.1 (6)	C25—C26—C27—C28	0.0 (7)
C8—C9—C10—C11	0.6 (6)	C26—C27—C28—C29	−0.6 (7)
C9—C10—C11—C12	−1.0 (6)	C27—C28—C29—C30	0.8 (7)
C10—C11—C12—C7	0.8 (6)	C26—C25—C30—C29	−0.2 (6)
C8—C7—C12—C11	−0.1 (6)	C24—C25—C30—C29	178.3 (4)
C6—C7—C12—C11	−179.8 (3)	C28—C29—C30—C25	−0.4 (6)
O1—C13—C14—N4	175.1 (3)	O2—C31—C32—N8	168.5 (3)
N3—C13—C14—N4	−5.0 (4)	N7—C31—C32—N8	−12.5 (5)
O1—C13—C14—C15	−4.8 (5)	O2—C31—C32—C33	−12.7 (5)
N3—C13—C14—C15	175.1 (3)	N7—C31—C32—C33	166.3 (3)
N4—C14—C15—C16	0.3 (5)	N8—C32—C33—C34	0.7 (5)
C13—C14—C15—C16	−179.8 (3)	C31—C32—C33—C34	−178.0 (3)
C14—C15—C16—C17	0.7 (5)	C32—C33—C34—C35	0.2 (5)
C15—C16—C17—C18	−0.9 (6)	C33—C34—C35—C36	−1.6 (6)
C16—C17—C18—N4	0.3 (6)	C34—C35—C36—N8	2.2 (6)
C2—C1—N1—C5	3.4 (5)	C20—C19—N5—C23	1.9 (5)
C2—C1—N1—Cd1	−162.4 (3)	C20—C19—N5—Cd2	−171.9 (3)
C4—C5—N1—C1	−3.3 (5)	C22—C23—N5—C19	−1.5 (5)
C6—C5—N1—C1	177.1 (3)	C24—C23—N5—C19	176.9 (3)
C4—C5—N1—Cd1	163.5 (3)	C22—C23—N5—Cd2	172.8 (3)
C6—C5—N1—Cd1	−16.2 (4)	C24—C23—N5—Cd2	−8.8 (4)
C7—C6—N2—N3	0.6 (5)	C23—C24—N6—N7	177.4 (3)
C5—C6—N2—N3	179.3 (3)	C25—C24—N6—N7	−0.9 (5)
C7—C6—N2—Cd1	−165.7 (3)	C23—C24—N6—Cd2	17.4 (4)
C5—C6—N2—Cd1	13.0 (4)	C25—C24—N6—Cd2	−160.9 (3)
O1—C13—N3—N2	−4.9 (5)	C24—N6—N7—C31	−175.4 (3)
C14—C13—N3—N2	175.2 (3)	Cd2—N6—N7—C31	−14.7 (4)
C6—N2—N3—C13	−179.2 (3)	O2—C31—N7—N6	−1.6 (5)
Cd1—N2—N3—C13	−12.2 (4)	C32—C31—N7—N6	179.4 (3)
C17—C18—N4—C14	0.6 (5)	C35—C36—N8—C32	−1.3 (6)
C15—C14—N4—C18	−0.9 (5)	C33—C32—N8—C36	−0.2 (5)
C13—C14—N4—C18	179.1 (3)	C31—C32—N8—C36	178.6 (3)
N3—C13—O1—Cd1	18.3 (4)	N7—C31—O2—Cd2	15.8 (4)
C14—C13—O1—Cd1	−161.9 (3)	C32—C31—O2—Cd2	−165.3 (3)

### Hydrogen-bond geometry (Å, º)

**Table:**

*D*—H···*A*	*D*—H	H···*A*	*D*···*A*	*D*—H···*A*
N3—H3···N4	0.88	2.27	2.629 (4)	104
N7—H7···N8	0.88	2.28	2.640 (4)	104
